# Racial and Ethnic Disparities in Research Studies: The Challenge of Creating More Diverse Cohorts

**DOI:** 10.1289/ehp.123-A297

**Published:** 2015-12-01

**Authors:** Lindsey Konkel

**Affiliations:** Lindsey Konkel is a New Jersey–based journalist who reports on science, health, and the environment. This reporting project was funded through a grant provided by the Reporting Award at New York University’s Arthur L. Carter Journalism Institute.

The shafts of midmorning sun that spill into San Francisco’s Visitacion Valley belie the dank gloom inside Crystal Paniagua’s home. The 27-year-old shows a community health worker where cockroaches have been coming in through light fixtures and electrical outlets in the dilapidated two-bedroom apartment she occupies with her mother and four kids in the Sunnydale–Velasco Projects—one of the poorest, most crime-ridden neighborhoods in San Francisco.

Paniagua’s son Darian, a shy seven-year-old with asthma and roach allergies, hangs back as she opens the black box where she keeps his medications—allergy pills, nasal decongestants, eczema cream, and an albuterol inhaler and spacer. “He’s been telling me for a few days that his nose is hurting,” says Paniagua. She explains to the community health worker that his cough gets worse at night. The medications do little to help ease his breathing, which sounds like whistling.

**Figure:**
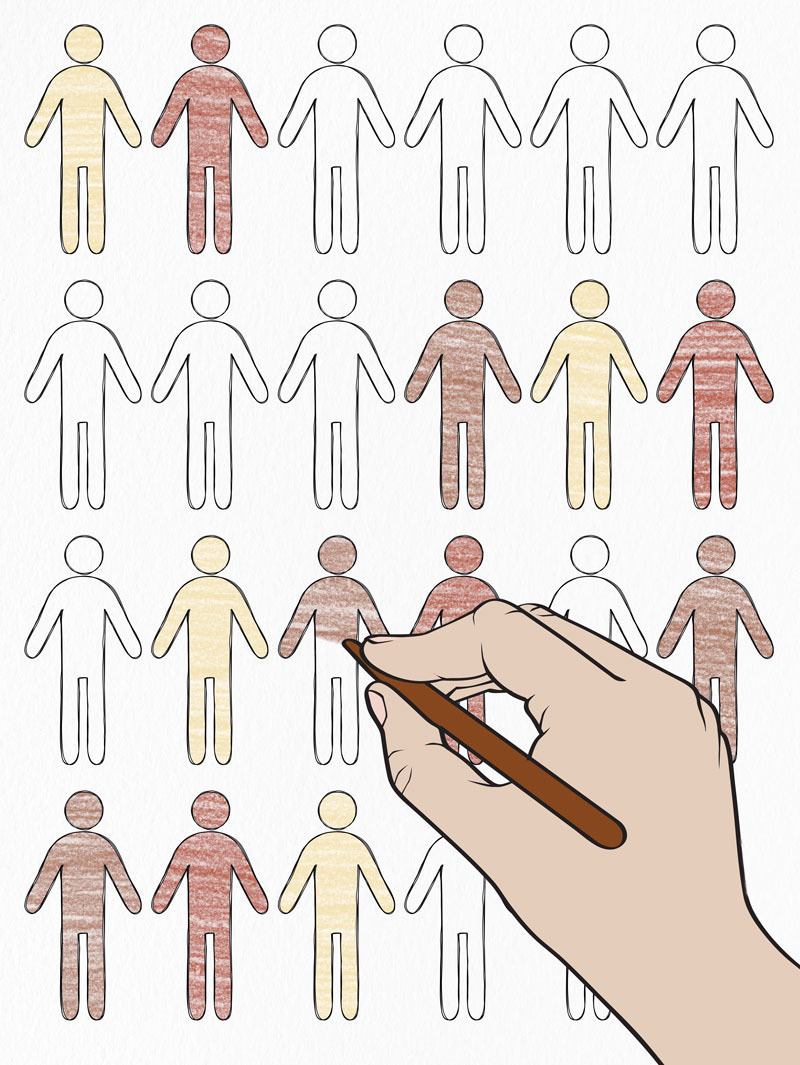
Minority populations are much less likely than their white counterparts to be included in studies on environmentally related diseases, even those that disproportionately affect minority communities. A failure to create more racially diverse research cohorts, some experts say, could exacerbate existing health disparities. Figures: © Natalia Sheinkin/Shutterstock; hand: © Cox Design/Shutterstock

Minority kids like Darian—his mother says he has Mexican, Puerto Rican, Dominican, and Samoan ancestry—are more likely to deal with pollution, poor housing, and asthma than their white counterparts.^1^ Yet people of color are less likely to be included in biomedical research studies to address asthma and other environmentally related diseases, such as cancer and diabetes, that disproportionately affect minority communities.^2^^,^^3^

The U.S. Census Bureau predicts that within 30 years the nonwhite proportion of the American population will shift to more than 50%.^4^ A failure to create more racially diverse research cohorts, some experts say, could exacerbate existing health disparities if those most affected by disease continue to be excluded. Others call it a missed scientific opportunity to fully understand the factors that lead to poor health and disease. Still others point to the high cost of racial and ethnic health disparities. By one estimate, reducing racial and ethnic health disparities would have saved the United States over $1.2 trillion in direct and indirect medical costs between 2003 and 2006 alone.^5^ New multidisciplinary studies examining the complex associations between genes, socioeconomics, and environmental exposures only underscore the need to include more minority participants in biomedical research.

## Defining the Problem

The 1993 National Institutes of Health (NIH) Revitalization Act mandates the inclusion of racial and ethnic minorities in federally funded biomedical research.^6^ However, fulfilling this mandate has proven problematic, even as evidence accumulates that race and ethnicity play an important role in disease risk as well as in responses to environmental exposures and drug therapies.

For instance, blacks and Latinos make up 30% of the U.S. population but account for just 6% of all participants in federally funded clinical trials.^3^ A review of government-funded cancer research studies found that all racial/ethnic minorities—including Asian Americans, Native Hawaiians, Pacific Islanders, American Indians, and more—are considerably underrepresented in cancer clinical trials, and fewer than 2% of these studies have focused on minority health needs.^6^ Similarly, fewer than 5% of federally funded lung disease studies in the last 20 years have focused on people of color,^2^ even though black Americans are one-third more likely than whites to have asthma and over three times more likely to die from it.^7^

“Minority communities shoulder a disproportionate share of the country’s environmental problems, yet there are major gaps in our understanding of how environmental exposures and health interact in these smaller subgroups,” says Symma Finn, a medical anthropologist and program officer for the Population Health Branch of the National Institute of Environmental Health Sciences. “Public health interventions that benefit the majority Caucasian population may not be applicable or may need to be adapted for use in communities that are most at risk of disease.”

There are several possible reasons why minorities are underrepresented in biomedical research studies. Cultural and linguistic differences as well as financial and time constraints—for example, not having the time and money to travel to a study site—may hinder minority participation. Researchers may be insufficiently trained to design and implement studies in minority communities or lack the incentive to recruit and retain sufficient numbers of participants. ^2^^,^^6^

Some experts believe that fear of exploitation, based on unethical practices that have been documented in past studies, may make minority communities distrustful of the biomedical establishment and reluctant to participate today.^8^ For example, in the Tuskegee Syphilis Study conducted between the 1930s and 1970s, black men with syphilis were denied proven treatments for the disease while researchers studied the effects of untreated syphilis on the body.^9^ More recently, investigators at Arizona State University collected blood samples from the Havasupai Tribe to study genetic markers of type 2 diabetes, but then used the samples for unrelated studies on schizophrenia and inbreeding—taboo topics for the Havasupai—without the consent of tribal members.^10^ (In an out-of-court settlement tribal members eventually received compensation of $700,000, funds for a clinic and school, and the return of their DNA samples.^10^)

Yet research shows that many individuals of racial and ethnic minorities are as willing to participate in research studies as whites when given the opportunity and when research objectives are translated into a culturally relevant context.^8^ “It’s not that minorities are hard to reach but that they’re hardly reached,” says Moon Chen, a cancer health disparities expert at the University of California, Davis.

Race and ethnicity are complex concepts that can offer important clues to researchers beyond one’s genetic risk. “You can infer a lot about a person’s socioeconomic status, their environmental exposures, diet, and other cultural considerations,” says Sam Oh, an epidemiologist in the Asthma Collaboratory, an asthma genetics lab at the University of California, San Francisco (UCSF).

Research suggests that when social factors, such as access to health services, are equalized, some racial disparities dissipate. In one study, researchers found that poor whites living in the racially integrated, low-income neighborhood of Southwest Baltimore had similar health risks as their black counterparts. The researchers found that nationally reported racial disparities in hypertension, diabetes, and obesity among women were either reduced or nondetectable among the Baltimore cohort.^11^

## A Tailored Approach to Treatment and Prevention

Researchers have made dramatic advances in understanding the genetic basis of many common diseases since the sequencing of the human genome was completed in 2003.^12^ Large public databases of genomic data allow for a closer look at health and disease at the molecular level.^13^ This “big data” approach is beginning to result in more targeted therapies for diseases, including so-called precision medicine, an approach to disease treatment and prevention that takes into account individuals’ variability in genes, environment, and lifestyle. In his 2015 State of the Union Address, President Barack Obama announced plans for the federal government’s $215 million Precision Medicine Initiative, which will include the creation of a research cohort of more than 1 million Americans.^14^

Determining who ultimately will benefit from the genomics revolution and new tailored approaches to disease treatment and prevention depends largely on who is studied, says Esteban Burchard, a physician scientist at the UCSF Asthma Collaboratory and a member of the Precision Medicine Initiative Working Group,^15^ which has advised the NIH on how to recruit study participants. In the past, racial and ethnic minorities have been largely excluded from genetic disease studies. As of 2011, 96% of the participants in the more than 1,000 genome-wide association studies conducted to that point were of European descent.^12^ Most physicians and scientists are therefore informed by research extrapolated from a largely white population, according to Burchard and colleagues.^3^

When minorities are included in biomedical research, they may be inappropriately aggregated into large groups, such as “Asian” or “Latino.” This is partly related to the problem of subgroup size—aggregation is necessary to get enough people in each subgroup to produce statistically significant results. But it comes at the cost of true subgroup homogeneity, and it masks the fact that severe health disparities often exist among racial and ethnic subgroups within the minority population.^16^

Cambodian and Hmong immigrants, for instance, experience poorer overall health outcomes than other Asian Americans.^16^ And while Asian Americans as an aggregated group appear to have similar rates of heart disease as whites, prevalence is much higher among Asian Indians and Filipinos and lower among Chinese, Japanese, and Korean Americans.^17^ “We end up extrapolating or generalizing that what works in one group will work for another, but that’s not always the case,” says Latha Palaniappan, an internist at Stanford School of Medicine who studies cardiovascular risk in Asian Americans.

Recent studies in the field of pharmacogenomics, which probes how genes affect a person’s response to a drug, show that a higher prevalence of certain genetic traits that influence drug metabolism may exist in some Asian-American subgroups. An estimated 75% of Pacific Islanders have a genetic trait that makes them respond poorly to the blood thinner clopidogrel, which puts them at risk of clotting and recurrent heart attacks.^18^ Up to 86% of people of Asian descent are estimated to be hypersensitive to warfarin, the most commonly used anticoagulant drug, increasing their risk of excessive bleeding at higher therapeutic doses.^19^

Capturing complex demographic origins of a variety of cultural groups will become increasingly important for biomedical and public health efforts in diverse populations, according to a team of researchers from the Icahn School of Medicine at Mount Sinai in New York. In a presentation at the American Society of Human Genetics 2015 annual meeting, the team described how they combined genetic data, ancestry information, and electronic health records from more than 31,000 New Yorkers to identify ultrafine-scale patterns of genetic diversity within the city.^20^ This approach, they say, can be applied to other cities around the world that are becoming as diverse as New York City.^21^

## Unraveling Racial Disparities in Asthma

While Esteban Burchard was an internal medicine resident at Brigham and Women’s Hospital in Boston in the late 1990s, a black teen died of an asthma attack, clutching his rescue inhaler, just blocks from the emergency room where Burchard worked. The fast-acting asthma medication had failed to relax the muscle spasms constricting the teen’s airways.

Burchard was working on an asthma genetics research project at the time. He discovered a variant in a gene encoding interleukin 4, a protein involved in inflammatory and immune responses that was predictive of more severe asthma in white people. This same gene variant, he found, was 40% more common among black asthmatics than whites.^22^

Asthma has one of the most striking racial disparities of any disease in the United States, Burchard says. Asthma prevalence among Puerto Rican and black children in the United States is double that of white kids.^23^^,^^24^ Mexican-American children generally have far less asthma, but those who do have it often face worse asthma-related outcomes (including missed school days and trips to the emergency room) than their white peers.^24^

When Burchard returned to his hometown of San Francisco a few years after his residency for a pulmonology fellowship at UCSF, he established cohorts to study how genetic and environmental risk factors associated with race and ethnicity influence asthma in black and Latino kids.^25^ Realizing he’d never be able to recruit a large sample of low-income minority children from academic research and referral centers, Burchard partnered with clinicians and community health workers across the country who had already established strong ties with patients. Combined, the cohorts represent more than 9,000 children with asthma from centers across the United States, Puerto Rico, and Mexico. They make up the largest gene–environment study of asthma in minority children in the United States.

Among their findings, Burchard and colleagues have shown that nearly half of all black children and two-thirds of all Puerto Rican children with moderate to severe asthma don’t respond to albuterol, the most commonly prescribed asthma medication.^26^ They also found that among Latinos, having a higher percentage of African ancestry was associated with higher odds of asthma and poorer lung function, while Native American ancestry appeared protective against asthma.^27^

## Integrating Genes and Environment

As a physician and a social epidemiologist, Neeta Thakur wants to find a way to better identify the asthma patients who would be most profoundly affected by social and environmental exposures. “I think it has become abundantly clear that complex diseases like asthma can’t simply be explained by studying just genetics or just social determinants,” says Thakur, who works with the UCSF Asthma Collaboratory.

Thakur uses genetics and bioinformatics techniques to address how social and environmental factors may modulate biology and disease susceptibility. “We know the burden of asthma is highest in socioeconomically disadvantaged communities,” she says, “and we know air pollution is bad.” But the impacts of socioeconomic stress^28^ and environmental exposures^29^ may vary among racial and ethnic subgroups.

In a 2013 study Thakur and colleagues found that for black kids, risk of asthma increases as socioeconomic status decreases.^30^ The trend was reversed for Mexican-American kids—the poorer they were, the less likely they were to have asthma. The findings in Mexican Americans may be due in part to acculturation, according to the authors—as an immigrant group becomes more assimilated into U.S. culture, they adopt less healthful behaviors, including poorer eating habits, more smoking, and reduced breastfeeding.

Thakur’s team is now examining how poverty interacts with biological markers of stress in black Americans to better understand how socioeconomic risk factors modify the course of asthma. “We want to identify the most susceptible individuals so that we can better develop targeted interventions for them,” Thakur says.

Determining the unique environmental and health challenges faced by racial and ethnic subgroups is another way to help target interventions. Take chronic kidney disease, for instance. Across the U.S. population, diabetes and heart disease are major risk factors for chronic kidney disease.^31^ Studies among the Navajo in the Southwest have found an additional, unexpected risk factor for this group—decades of exposure to uranium mining activities and drinking water contaminated by mine waste.^32^^,^^33^ Interventions to reduce environmental exposures may be more suitable than pushing blood pressure–lowering drugs, says Johnnye Lewis, a toxicologist at the University of New Mexico and director of the Navajo Uranium Assessment and Kidney Health Project.

Developing effective and culturally relevant interventions means asking culturally relevant questions. For Lewis, that meant listening to the concerns of community members and brainstorming with Navajo leaders who wanted to know whether exposure to toxic mine waste piles on their lands could be responsible for their high rates of kidney disease.

Her research is supported by the newly funded NIH–U.S. Environmental Protection Agency Centers of Excellence on Environmental Health Disparities Research, which aim to address modifiable risk factors contributing to health disparities.^34^ Other research studies supported by the centers will investigate how environmental exposures contribute to obesity in Latina women, how living in poor-quality housing affects health outcomes in a black cohort, and the combined effects of poverty and air pollution on residents of rural Appalachia and urban Baltimore.

Studies supported by the centers will focus on engaging members of affected communities in the research and asking environmental health questions with direct relevance to these study participants, says Finn. “The people participating in the studies are entrusting researchers with very valuable personal health information,” she says. “A contribution to scientific knowledge isn’t enough—the community needs to see concrete benefits, too.”

## Subtle Biases

Yet for researchers who want to address health questions in minority populations, challenges remain. For instance, while the 1993 NIH Revitalization Act mandated minority inclusion, few mechanisms exist to enforce inclusion policies, says Eliseo Pérez-Stable, director of the National Institute on Minority Health and Health Disparities. This institute was established in 2010 under the Affordable Care Act to investigate scientific questions surrounding health disparities.^34^

Unseen racial biases can permeate medical decision making and research peer-review processes, influencing who gets recruited into studies.^35^ “Even scientists, some of the most objective people in the world, are susceptible to subtle prejudices you wouldn’t consciously recognize as racial bias,” says John Dovidio, a psychologist at Yale University who studies this issue.

These subtle biases can lead to exclusion. For instance, says Dovidio, physicians or researchers may be less likely to mention or recommend a certain study to patients who they assume would not want to participate anyway. Or, they may unconsciously overemphasize negative outcomes when discussing a study with a minority patient, which may deter the patient from participating.

Subtle and unexamined racial biases can affect decisions over research funding, too. Minority researchers and doctors may be more likely to focus on health disparities and minority populations^36^ yet less likely to receive federal funding for their research. In one analysis, white scientists were twice as likely as their black counterparts to receive NIH grants for their research.^37^ The authors of the analysis controlled for variables such as education, training, and experience, yet their models could not explain why black investigators were less likely to be funded than white investigators.

“We find it troubling that the typical measures of scientific achievement—NIH training, previous grants, publications, and citations—do not translate to the same level of application success across race and ethnic groups,” the study authors wrote.^37^

These findings helped prompt a multi-year NIH probe to determine whether unconscious racial biases impede minority researchers from receiving funding and to detect and correct racial bias in the grant-making peer-review process.^38^ In 2014 the NIH Center for Scientific Review announced a challenge for investigators to devise new methods to detect bias in peer review and strengthen impartiality.^39^ According to a press release announcing the winners, many of the entries overlapped with ideas NIH is already considering, which center director Richard Nakamura said “suggests we are on the right track.”^40^

## Challenges of Addressing Institutional Bias

Tackling bias in the federal grant-making process is critical to reducing racial disparities among both researchers and study participants, says Dovidio, who is also a member of NIH’s Diversity Working Group Subcommittee on Peer Review. But unintentional bias is complex and subtle, making it difficult to quickly eliminate.

The disparity in funding between white and nonwhite applicants has remained largely unchanged in the past three decades. UCSF epidemiologist Sam Oh found that in 2013, 23.3% of white applicants for certain NIH grants received funding compared with 19.3% of nonwhite applicants. In 1985 those numbers were 48.6% and 42.1% for white versus nonwhite applicants.^3^ “The big takeaway,” Oh says, “is that white and nonwhite scientists have been getting funded at systematically different rates for the past three decades.”

Increasing the diversity of grant reviewers may be one solution. Underrepresented minorities made up 10.9% of NIH study reviewers in 2013, up only marginally from 10% in 2000.^3^ “Unintentional biases don’t operate as much in diverse groups,” says Dovidio. He points to research on jury deliberations that suggests having more diversity in a group of decision makers can result in more thoughtful and better-informed decisions.^41^

But in some respects, making this change is easier said than done. Reviewers are chosen from the pool of successful grant applicants.^3^ If minority researchers are less likely to win grants, the problem is self-perpetuating, Oh says.

However, initiatives such as the NIH Early Career Reviewer Program, which provides opportunities for early-stage researchers to serve on review panels, could help to even the numbers. A third of researchers accepted into the program are individuals from underrepresented groups.^42^

While there may be no quick fix to disparities in the federal grant-making process, there are highly feasible changes that can ensure minority participants are being adequately represented in research now, says Oh. Solutions might include positioning study sites in areas with diverse residents, employing recruitment staff with whom participants can communicate in their own language, providing travel support for participants who lack access to transit, and creating culturally sensitive informational materials about how data will be collected and used.^3^

From a policy perspective, says Oh, the NIH could use as a model for enforcement a recent mandate to require sex and gender inclusion plans in preclinical research.^43^ “By studying diverse populations, you do better science,” he says. “It’s a worthwhile investment scientifically.”
